# Comparison of Three Different DNA Extraction Methods for *Linguatula serrata* as a Food Born Pathogen

**Published:** 2017

**Authors:** Gilda ESLAMI, Sepideh KHALATBARI-LIMAKI, Mohammad Hasan EHRAMPOUSH, Mostafa GHOLAMREZAEI, Bahador HAJIMOHAMMADI, Ahmad ORYAN

**Affiliations:** 1. Research Center for Food Hygiene and Safety, Shahid Sadoughi University of Medical Sciences, Yazd, Iran; 2. Dept. of Parasitology and Mycology, Faculty of Medicine, Shahid Sadoughi University of Medical Sciences, Yazd, Iran; 3. Dept. of Food Hygiene and Safety, Faculty of Health, Shahid Sadoughi University of Medical Sciences, Yazd, Iran; 4. Dept. of Environmental Health, Faculty of Health, Shahid Sadoughi University of Medical Sciences, Yazd, Iran; 5. Dept. of Pathology, School of Veterinary Medicine, Shiraz University, Shiraz, Iran

**Keywords:** DNA, Genomics, Pentastomida, Food safety

## Abstract

**Background:**

One of the most important items in molecular characterization of food-borne pathogens is high quality genomic DNA. In this study, we investigated three protocols and compared their simplicity, duration and costs for extracting genomic DNA from *Linguatula serrata*.

**Methods:**

The larvae were collected from the sheep’s visceral organs from the Yazd Slaughterhouse during May 2013. DNA extraction was done in three different methods, including commercial DNA extraction kit, Phenol Chloroform Isoamylalcohol (PCI), and salting out. Extracted DNA in each method was assessed for quantity and quality using spectrophotometery and agarose gel electrophoresis, respectively.

**Results:**

The less duration was regarding to commercial DNA extraction kit and then salting out protocol. The cost benefit one was salting out and then PCI method. The best quantity was regarding to PCI with 72.20±29.20 ng/μl, and purity of OD260/OD280 in 1.76±0.947. Agarose gel electrophoresis for assessing the quality found all the same.

**Conclusion:**

Salting out is introduced as the best method for DNA extraction from *L. seratta* as a food-borne pathogen with the least costand appropriate purity. Although, the best purity was regarding to PCI but PCI is not safe as salting out. In addition, the duration of salting out was less than PCI. The least duration was seen in commercial DNA extraction kit, but it is expensive and therefore is not recommended for developing countries where consumption of offal is common.

## Introduction

Molecular detection and identification of any organism especially food-borne pathogens requires extracted high quality DNA. There are various documents regarding to protocols for DNA extraction from different eukaryotes especially arthropods, but based on our knowledge, there is no reports for a simple and safe methods in *Linguatula serrata* larvae as a Pentastomida (1)*.* This parasite is a food-borne pathogen with a shape similar to tongue, the reason that it is named tongue worm (2–4). Canids are considered as its definitive host. Animals such as sheep, goat, camel, and cattle as the intermediate host infect with swallowing the eggs containing larvae (2, 5). The larvae inside the egg release in intestine and then colonize in internal tissues such as mesenteric lymph nodes (MLNs), liver, and lung, for developing to numph (6–10). Human is infected by consumption of raw or semi raw infected edible organ of herbivores, that known as Halzon syndrome. It is prevalence in Middle East countries because of their habitation in eating of edible offal of domestic in raw or semi raw manner (11, 12).

To date, themorphology and biological characters use as common methods for detection and identification of *L. serrate* (12–15). There are only a few studies so far that have been subjected the molecular characterization of genomic and/or mitochondrial DNA. Although, there is some evidences that have suggested a close relationship between Pentastomida and the crustacean subclass Branchiura (16–21), the taxonomic rank and systematic position of them have not fixed yet (14, 15). It seems that introducing a DNA extraction protocol, as the important stage for molecular studies with handy, simple, and cost benefit characters could be helpful. This parasite has a hard tegument and therefore, common methods are not appropriate for DNA extraction from this food-borne pathogen (1).

In this study, the modified Phenol Chloroform Isoamyl alcohol (PCI) and salting out methods were designed for DNA extraction from *L. seratta* and then the extracted DNA were compared with the one resulted from commercial DNA extraction kit.

## Materials and Methods

### Sample collection and preparation

During May 2013, MLNs were obtained from slaughtered sheep in the Yazd Slaughterhouse, immediately transferred to the laboratory. The nymphs were then collected by longitudinal sectioning of the MLNs and immersing in a glass petri dish containing sterile Phosphate Buffer Saline (PBS) for about 5–10 min (8). They were washed double in sterile PBS. The isolated nymphs were stored in 70% ethanol and kept at −20°C for next steps.

The study was approved by Ethics Committee of the university.

### DNA extraction

DNA extraction was performed based on three techniques, including PCI, salting out, and commercial kit that are explained as below. For each group, five nymphs were considered and the examinations were repeated in triple.

### DNA extraction with PCI method

This protocol was performed based on the modified method recommended by Sambrook and Russel (22). After washing the nymphs for three times with PBS, two groups were designed in this section each containing five nymphs. The first and second groups named PCI_1_ and PCI_2_ that were lysed with NET (NaCl, 50 mM; EDTA pH 8, 25 mM; Tris-HCl pH 7.6, 50 mM) buffer and the ready lysis buffer inside the DNA extraction kit (Qiagen #69504), respectively. Each type of lysis buffer (500 μl) was added to each tube containing five nymphs. In PCI_1_, SDS was added to a final concentration of 1%. RNase A (Thermo Scientific, Massachusetts, USA) was added to a final concentration of 100 μg/ml. The samples were incubated at 37 °C for 30 min. Proteinase K (Thermo Scientific, Massachusetts, USA; 20 μl of a 10 mg/ml solution) was added to a final concentration of 400 μg/ml. The samples were incubated at 60 °C for an overnight inside the rotation hybridization oven. For PCI_2_ group, the lysis buffer of the kit was used according to the manufacturer’s instruction. Consequently, equal volumes of equilibrated phenol (Cinnagen, Tehran, Iran) was added to each PCI_1_ and PCI_2_ sample (500 μl), and mixed gently for 5 min. After centrifugation at high speed (14000 xg) for 5 min at room temperature, the upper phase was carefully removed and transferred to a new sterile 1.5 ml microtube. A mixture of phenol, chloroform, and isoamyl alcohol (25:24:1) was added in equal volumes to each sample. The samples were mixed gently and centrifuged for 3 min. The upper phase was again transferred to a new sterile 1.5 ml microtube. Equal volume of chloroform was added and each sample was centrifuged for 1 min. The upper phase was once again transferred to a new sterile 1.5 ml microtube. The DNA samples in both groups were precipitated, using 50 μl of 3 M sodium acetate (NaAc 300 mM, pH 6.0) and at least twovolumes of cold (−20 °C) ethanol and kept on ice for 30 min. Subsequently, the samples were centrifuged at 14000 xg for 10 min at 4 °C. Ethanol was removed and each DNA pellets was washed with cold (−20 °C) 70% ethanol (250 μl). The samples were centrifuged at 14000 xg for 10 min. The ethanol was removed, and the DNA pellet was dried. The DNA sample was then resuspended with appropriate amount of double distilled water or TE buffer (100 μl) and incubated 30 min at 56 °C. The DNA was aliquot and stored at −20 °C for next analysis.

### DNA extraction with salting out method

This protocol was performed based on the modified method recommended by Sambrook and Russel (22). After washing the nymphs three times with PBS, two groups were designed in this section, each containing five nymphs. The first and second groups named S_1_ and S_2_ and were lysed with NET (NaCl, 50 mM; EDTA pH 8, 25 mM; Tris-HCL pH 7.6, 50 mM) buffer and the ready lysis buffer inside the DNA extraction kit (Qiagen #69504), respectively. Each type of lysis buffer (500 μl) was added to each tube containing five nymphs. In S_1_, SDS was added to a final concentration of 1%. RNAse was added to a final concentration of 100 μg/ml and the samples were incubated at 37 °C for 30 min. Proteinase K (20 μl of a 10 mg/ml solution) was added to a final concentration of 400 μg/ml. The samples were digested for an overnight and rotated in a hybridization oven at 60 °C. For S_2_, the lysis buffer of the kit was used according to the manufacturer’s instruction. Consequently, for both groups of S_1_ and S_2_, purification of DNA was done by adding 250 μl NaCl 6 M. After centrifugation, the supernatant was removed and added to a new sterile 1.5 ml microtube and precipitated with cold absolute ethanol. After washing with ethanol 70%, the pellet was diluted in 100 μl ddH_2_O and stored at − 20 °C until examination time.

### DNA extraction with kit

One group named group K was selected for DNA extraction with commercial kit (DNA extraction kit, Bioneer, Korea). This method was done based on the manufacturer’s instruction.

### Analysis of DNA

The DNA concentration was detected using spectrophotpmeter at absorption of 230, 260, 280 and 320 nm. The ratio of OD260/OD280 was calculated for estimation of the purity of the extracted DNA. In addition, its quantification analyzing was performed using 0.8% agarose gel electrophoresis and visualized using gel documentation (E-Gel® Imager, life technologies, USA).

### Statistical analysis

All experiments were carried out in triplicate. ANOVA test was applied to perform statistical analysis of data, using SPSS 16.0 software (Chicago, IL, USA). Differences between means were considered statistically significant at the 95% confidence level (*P*<0.05).

## Results

**Fig. 1:**
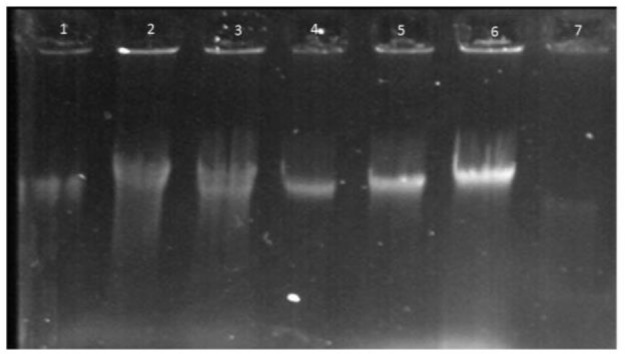
Agarose gel electrophoresis (0.8%) of the genomic DNA. Lane 1&2: PCI_2_ method; Lane 3&4: PCI_1_ method; Lane 5: S_2_ method; Lane 6: S_1_ method; Lane 7: K method

After sampling, the genomic DNA was extracted with three main different methods as explained in material and method section. For analyzing, the quality and quantity was measured using agarose gel electrophoresis (0.8% agarose) and spectrophotometer, respectively. Analysis of extracted genomic DNA showed that the less duration was regarding to commercial DNA extraction kit and then salting out protocol. The cost benefit one was salting out and then PCI method. The efficiency of DNA extraction by these methods is illustrated in Table 1 and Fig. 1.

**Table 1:** Efficiency of the present DNA extraction method as determined by spectrophotometry at 230, 260, 280 and 320 nm. values are mean±standard deviation of six measurements

**Methods**	**DNA concentration (ng/μl)**	**A_230_**	**A_260_**	**A_280_**	**A_320_**	**A260/280**
PCI_1_	29.37±2.84	0.075±0.024	0.006±0.096	0.038±0.015	0.013±0.002	1.76±0.947
PCI_2_	72.20±29.20	0.707±0.205	0.058±0.006	0.015±0.001	0.016±0.003	1.96±0.419
S_1_	48.13±9.91	0.211±0.077	0.155±0.081	0.086±0.037	0.058±0.012	1.68±0.048
S_2_	111.18±41.91	0.819±0.551	0.222±0.084	0.204±0.097	0.158±0.237	1.82±0.295
K	14.56±3.06	0.048±0.011	0.031±0.006	0.007±0.006	0.031±0.005	1.86±2.332

Briefly, the mean concentration of extracted DNA in PCI_1_ and PCI_2_ was 29.37±2.84 ng/μl and 72.20±29.20 ng/μl, and their mean ratio of A (260)/(280) were 1.76±0.947 and 1.96±0.419, respectively. The extracted DNA concentration for S_1_ and S_2_ were 48.13±9.91 ng/μl and 111.18±41.91 ng/μl, and their ratio was 1.68±0.048 and 1.82±0.295, respectively. These data for K group were 14.56±3.06 ng/μl and 1.86±2.332. As shown in Fig. 1, the analysis of the extracted DNA on 0.8% agarose gel electrophoresis appeared high quality due to the single and pure band.

## Discussion

Genomic DNA analysis of the parasites with hard covering such as *L. serrata* needs appropriate techniques to yield pure DNA for molecular studies (23, 25). *L. serrata* is a metazoan with a thick and strong body. Besides the commercial kits that are very expensive especially for developing countries with low income, we are looking for safe and inexpensive methods for recovering the genomic DNA from this parasite with appropriate yields. In this study, the extracted DNA from *L. serrata* was done by three different methods and the results were compared in quantity and quality. As explained above, each group of PCI and S was divided in two subgroups (PCI_1_ and PCI_2_; S_1_ and S_2_). The yield of extracted DNA from each method was analyzed using spectrophotometer and agarose gel electrophoresis 0.8%.

The concentration of extracted DNA was analyzed using spectrophotometer. The best result was obtained from S_2_ with 111.18±41.91 ng/μl, which was one of the subgroup of S main method. In S_2_, the lysis buffer was the same as the one in commercial DNA extraction kit. This buffer contained chaotropic salt. These kinds of molecule are the main part of lysis buffer in different commercial kits resulted in stability of macromolecules such as nucleic acids by disruption of hydrogen bonding network inside the water molecules (26). In addition, these molecules denature the proteins especially in high concentration by reducing the degree of amino acids organization inside the protein structures (27). One of the strongest chaotropic salts is guanidinium chloride that is usually used in lysis buffer in different commercial DNA extraction kits. These materials are more effective than the normal lysis buffer that we used in the present study, which contained NaCl, 50 mM; EDTA pH 8, 25 mM; Tris-HCL pH 7.6, 50 mM.

Purity of the extracted DNA is another important variable in this process. In this study, the best purity was related to K and S_2_ groups with 1.86±2.332 and 1.86±2.332, respectively. The K group included method based on the commercial DNA extraction kit with using specific column for purification. In this method, extracted DNA bind to the silica that has been replaced the bottom of the microtube. This reaction would be accelerating using chaotropic agents by disruption of nucleic acid and water association. There are some other detergents in lysis buffer that facilitate protein solubilization and lysis in parallel of chaotropic agents. In the next step, during the washing, salt and polysaccharides pass through the silica. Therefore, extracted DNA would have the appropriate purity.

In S_2_ group, purity was appropriate, too. In this method, we used the lysis buffer of kit but without silica column. Salting out method could recover the genomic DNA with high purity. This study showed that S_1_ with traditional process had the least purity comparison with other methods that is agree with some other studies (28–30). However, the other methods showed appropriate purity that would be considered for PCR based techniques. The PCI_1_was the best method for removing salts from the extracted DNA because the least absorbance was seen in 230 nm in comparison with other methods, which is verified with some other studies (31, 32). Overall, there was no difference in concentration of extracted DNA between S_2_ and PCI_2_ methods (*P*>0.05). As shown in Fig. 1, all methods could extract DNA from this parasite with high quality because all showed a single band without any smear.

## Conclusion

DNA analysis of *L. serrata* needs an appropriate DNA extraction. Our study showed non-commercial DNA extraction methods using modified PCI and salting out could be useful because of no expensive and its safety especially salting out.
